# Address at the Commencement of the Dental College, at Ann Arbor, March 24th, 1880

**Published:** 1880-05

**Authors:** J. A. Robinson


### THE DENTAL REGISTER.

VOL. XXXIV.] MAY, 1880. [No. 5.

### ADDRESS
### AT THE
### COMMENCEMENT OF THE DENTAL COLLEGE,
### AT
### ANN ARBOR,

*March 24th, 1880.*

BY J. A. ROBINSON, D. D. S.

I remember when I was a boy of reading over and over again in an old school book until I had committed it to memory, a story of "A Journey of a Day—a Picture of Human Life," that began as follows:

"Obida, the son of Abensina, left the caravansary early in the morning and pursued his journey through the plains of Indostan; he was fresh and vigorous with rest; he was animated with hope; he was incited by desire; he walked swiftly forward over the valleys and saw the hills gradually rising before him. As he passed along his ears were delighted with the morning song of the bird of paradise, he was fanned by the last flutter of the sinking breeze, and sprinkled with dew by groves and spices; all his senses were gratified, and all care was banished from his heart." How well I recollect the delightful satisfaction I felt in the contemplation of such a journey; how I took up the map and found the Peninsula of Indostan, and what a desire I had to sail to Calcutta and take such a journey as I found described in that old school book.

I never dreamed that the beautiful Allegory by Dr. Johnson, could be any thing but a true picture. I never once then thought if I sailed to Calcutta that I should have to cross the sea that was full of waves; that as I mounted one of those waves it was only to go into the hollow of another; that they would be endless in number until I reached my destination. I did not think then that the storms might come that would pile them one upon the other into mountain heights, that would have to be overcome before I reached the end of my journey, or that perhaps such a calm would come over the sea as is described by Coleridge, where he says: "the very sea did rot," which is worse than any storm in human life; I did not think that this life was an ascent to a high mountain of excellence and character, and that if we rise at all, we must *climb*. But as I grew older, I was forced to learn that if I would succeed in life, I *must climb*, that a mountain was before me; that I must struggle to reach the top if I would arrive at a high degree of excellence in my profession, for there were scores of climbers all around me with brave hearts and determined wills, all striving to reach its summit.

Ruskin tells us there are three great offices that mountain ranges are appointed to fulfill, to preserve health and increase the happiness of mankind. The first is to give motion to water, so that men can build their cities in the midst of fields that will always be fertile, in order to preserve the health and to establish lines of commerce on streams that cannot fail. The second is to maintain a constant change in the currents of air, they divide the earth into climates and cause perpetual currents of air to traverse their passes, to ascend or descend their ravines, altering both the nature and temperature of air in a thousand ways; beating it hither and thither in the pools of their torrents; closing it within clefts and caves where the sunbeams never reach, till it is as cold as November mists, then sending it forth again to breath softly across velvet fields, or to be scorched among sunburnt shales and shapeless crags; then drawing it back in moaning swirls through clefts of ice, and up into dewy wreaths above the snow fields; then piercing it with strange electric darts and flashes of mountain fire, and tossing high in fantastic storm clouds, as the dried grass is tossed by the mower, only suffering to depart at last when chastened and pure, to refresh the faded and far off plains

The third is to cause perpetual change in the soils of the earth so they will not become exhausted.

Now, it must be apparent to all of you that the studies you have been pursuing the past years have made you stronger to overcome the obstacles in life, than so many years of idleness and luxury could possibly have done, and there is a correspondence between the struggles in gaining an education and in climbing to the top of a mountain. Mountains fill us with awe as we contemplate their rugged sides and remember the weary hours and tired limbs and heart aches we undergo in pursuing our journey to the summit. So it is of a profession; but once having pushed into the pathway those terrors disappear, and although we find clouds and storms in the ascent, by hope and perseverance we see the blue beyond the darkness and mist, and sunlight and stars will guide us onward and upward. While the pure waters that run down its mountain sides will cheer and refresh us like beautiful thoughts; the balsam and the pines will invigorate and purify us like intellectual pursuits, the iron and the herbs will renew and vivify our blood as culture renews and vivifies the affections, while the gold and the silver mines will enrich and reward us like the true treasures we find in the mountains of learning as we climb on in the studies of our profession, and will enable us to overcome all obstacles in the daily duties of life.

The profession you have chosen, young friends, is a mountain of religion, of morals, of science, of history and of mechanics. Of religion, for we are helping to keep in order and repair the mill with which we grind the food to sustain life: and so are helping our fellows, which is simply helping our Creator. He, who in any manner helps to relieve pain and lighten the load of care and anxiety is a religious man, though he may have no formulated creed; or, been blessed by the laying on of hands in any church.

Of morals, for if we are true, we shall be thorough and just, which is the foundation of all morals in human life. Of science, for dentistry is based upon absolute scientific principles without even guess work, equivocation or doubting. Of mechanics, for good dentistry is mechanism of the finer and higher sort that stretches into art nearer than most of the learned professions, and although our history may not reach back as far into the centuries as medicine: if we judge the history of a thing by the progress it has made in benefitting mankind ; ours is by no means *insignificant.* Our profession has grown out of the necessities of our civilization. All reforms and improvements grow in that way. The old turn-key and the old-fashioned fire place have passed away ; they were the primitive methods to relieve suffering and pain.

The forceps came along like the stove, a more useful, beautiful and modern invention. And why may we not presume as time rolls away, that there will no longer be any use for these things of the present. That the world may be lighted and heated with some power yet unknown, that will far surpass the Electric Light? And that some agent will yet be discovered that will prevent the decay in tooth-bone; or at least prevent extraction; for we have already marched far in the direction of that desired object. We live in a democracy, and by democracy I mean where all questions are decided by the votes of the people. Formerly the people belonged to a king; now people belong to themselves. The professors of Edinburgh College whose fame has spread throughout the world were chosen by the people, by the mechanics and tradesmen, like our Regents; and their success lay in the fact, that they always tried to select the best men.

Now, while this is true, let us remember, that the more we are endowed with liberty and education, the greater is the responsibility. The more liberty we possess the more we owe, if we would be really free. In the centuries that have passed, all sculptors and painters and poets were patroned by nobility. The music was grander, the sculpture and paintings more exquisitely finished than any we find to-day, but it was only confined to the few. If we have now no Messiah's of Handel, or sculpture and paintings like those of Angelo and Raphael; we have the music for the millions : and every boot black can sing, and the news boys draw their charcoal sketches on the fences and walls as they journey to their daily toil and labor.

Carlisle may sneer at philanthropy and benevolence and Ruskin may long for the old days of the stage coach to come back and take the place of the railroad, but they will not come again; for our tree of civilization and learning has spread its shade all over the world and we have the microscope to teach us its rootlets that supply the nourishment to its overspreading branches that protect and shade and spread contentment over all the land. With all our blunders and mistakes it is better to trust the people than kings. The masses correspond to the age in which they live. There is an inspiration that belongs to every age: and intellect is the inspiration to-day.

No miracle is necessary to raise humanity to its highest degree of excellence. Our religion will keep pace with our democracy, for we have the religious faculty. I trust the day is not far distant when a curriculum will be supplied in every department of learning for our spiritual natures as well as for our intellectual: and we shall have the spiritual telegraph and the microscope, to help us in that direction as well as in science and physics.

One great advantage in climbing a mountain, lays in the fact that we are obliged to go slow: we must proceed with caution and examine every foot hold as we go forward step by step: and while we may be obliged to catch hold of the twigs or the rocks, and our hands may become sore in the scramble: it is far better than to try and go *too fast* and fall down: for the great law of gravitation is always in full force, and we cannot escape punishment for every careless step and shall be severely bruised for every neglect of duty: and this you will find true in the practice of your profession.

The frequent failures in filling teeth grow out of our lack of care in manipulation: The cavities are not well cleaned and hermetically sealed. Make haste slowly is a good motto.

The perfect flower is not produced until every thing is ready. The perfect fruit does not ripen until nature has turned every force with the greatest care and energy towards its perfection. It is said of Daniel Webster that after he removed to Boston he went through with all the classics that he had neglected or passed hurriedly over in College, to fit himself for the station he was called upon to fill in his profession; and in political life to which he had an aspiration.

The man who climbs does not have to be placed in position; he goes naturally to his seat as a Governor. It may take years of patient toil: but a well rounded life of perseverance and study is always sure of a call to position in this great needful world.

Do not think friends, because you have received a diploma, that all climbing is at an end. Your race in life has just begun if you expect to climb to a high seat.

Contrast your opportunities to-day with those of us who began nearly half a century ago, when there was not an instrument but the dentist was obliged to make with his own hands; with the simple text book "Bell on the teeth" within our reach: with no Colleges: and looked upon by the public generally and physicians particularly as though we had no "rights they were bound to respect;" and ask yourselves if we have not had to climb to keep with those who have had such superior advantages, in Colleges provided free and by a great State.

In those days the profession had to be souled by earnest men. Men who had to climb and it is impossible to succeed in any other way. We cannot rise without labor any more than we could climb a mountain by sitting down quietly at its base and admiring its green and beautiful slopes. But we must do more than this; we must be filled with enthusiasm enough to help those who are trying and not so far advanced as ourselves. We must adore the perseverance of all our competitors; and pity and despise the laziness of those who desire to live a life of idleness and luxurious ease.

Enthusiasm will make your labors blossom into life with your operations, and bear fruit at the end of every days toil. It is for you to decide whether you will be buffoons and stage players, or artists and heroes; and do not deceive yourselves, men are always the worst cheated who cheat themselves, for what you gain by deception, you lose in manhood. Every thing grows by reaction; the most pernicious of all weeds are the weeds of deception, for they blossom and bear fruit in the garden of the heart. Civilization must be balanced with morality or the education of the masses will be worse than useless. There is said to be more scepticism in the world to-day than in any former period of our history. And the free schools with their inductive method of reasoning are said to be the chief cause of it. The Baconian system of reasoning has nearly put an end to monarchies and nobilities—and this is only because the children of the nineteenth century are taught to think for themselves. Give the boys and girls a slate and a pencil and you make a republican form of government. When you learn pathology in its fullest sense, which is to find out something for yourselves, you put an end to all scholastic reasoning which is founded on authority without questioning, and you lay the corner stone of the civilization and freedom of the race. When you apply your therapeutics and histology and anatomy in their highest sense to life, you will find we are a complex machine. That this human organism has the religious faculty as strong as any one, perhaps the strongest we possess. Suppress it to-day and it will assert itself to-morrow. It has made the music and songs, the masses and oratorios of the ages. It has built the cathedrals the monasteries and churches in all christian lands; and sent pilgrims and worshippers from Abraham to Paul, and down through all succeeding generations. It has grown dentistry into one of the learned profession, and professional men have always been leaders in thought in every age of the world, and this will be the apology (if I need any) of talking to you at this time of the responsibility of your calling. Let me beseech you, my young friends, when you receive your diploma, never to barter it away for a trade; as trade is a barter, but a profession is supposed to be a cure for some evil that exists among the people of the body politic. Stand by the merits of your calling and you have nothing to fear. Draper tells us, that the age corresponds to the childhood, youth and manhood of humanity.

If that be true, there must be an old age and a decay, but no age or nation has gone to decay except through immorality. Some may say that strength lays in the power of the artillery, but in our late war it was the moral force that lay back of the artillery that gave us success. We sometimes think because we have built a railroad across the Rocky Mountains to the Pacific, we have accomplished all that needs to be accomplished, but there is an old saying that reads, "he that ruleth his spirit, is greater than he who taketh a city." Do not be afraid to climb by inductive reason. There is no conflict between religion and science. The conflict is between science and theology.

The voice of the Lord spake to the man of old, when he said, "come let us reason together, though your sins be as scarlet, they shall be white as snow, though they be red like crimson, they shall be as wool."

This was deductive reason, because it came from the *in* and reasoned to the *out*. The religion of Jesus came by deductive insight, and it was the religion of the man of Nazareth that built this institution. It is the religion of Nazareth that has built the asylums for the feeble minded, the blind and the insane, and provides for so many widows and orphans all over the land. We do not make truth, we find out truth; we do not make laws, we discover laws, and it is because these laws are so little understood that our mistakes occur, when wholesome laws have a state of fixedness in our hearts and minds, they will form an effectual prohibition society against all manner of intemperance.

Tobacco will be again turned into food for worms, and all intoxicants banished from civilized society. If you are unclean the *bacteria* that destroys the enamel of your teeth, and which you cannot see, and it is the most indestructable of all human organisms, will eat into your heart, destroy its effections, and leave you old while you are yet young, and worthless in the age in which you live. By the inductive method we extort from nature, we fairly *wrench* the truths from her, while by the deductive method it is a free gift and given to us like our life.

But if you are true to the principles you have received and live according to your privileges, your lives will break forth in songs in the morning as you pursue your profession, that will follow all the day long and keep watch over you with the stars at night, or when the clouds cover you with thick darkness and sometimes with sorrowful years. By doing this you follow nature in its highest sense of revelation and example.

Climb, says the tulip and the grass when it is born in the spring, and peeps its head from under the snow for blossom and fruitage. Climb, sing the birds of the morning as they fly high in the air, while the dew washes their wings, and they prepare for the work of each day. Climb, says the eagle as he sits upon the topmost cliff of the rock that overlooks all the valleys below, while his scream shall be music as it sounds through the hills and seems to say: climb up hither young man! climb up hither young maiden and be blessed, and I will show you all beneath you, and you shall have eyes with which you can look and behold the sun.

The office of every profession is to *cure*: to keep alive and in health that which is going to decay. The first desire given to life is to *live;* and this is dual in the desire for the life of the race. The first great nerve center of the brain of which man has three, while some of the lower order of animals have two, and some not more than one, goes to prove that fact.

Our profession is nearer the circumference than the physician, and hence has commanded less attention from the world and become established latter than the doctor of medicine; but if we are to remain perfect as a divine whole, while our office is more remote from the seat of life, it is of great importance. We are the advance guard of the army, we are the skirmishers and sharp shooters to destroy the enemies of the race. And what would you say of an advance guard, who would destroy one of his helpers? a sharp shooter who would take the life of his comrade? And this I consider a parallel with almost all tooth extraction. The great warfare must be turned against tooth extraction. Tooth extractors are parasites that prey upon the body politic. The salvation of our profession depends upon this thing. You may build a fort for protection like false teeth; but the day of walled cities is a thing of the past, and we cannot denounce in too unmeasured terms this wholesale extraction of the teeth. It is the barbarism of the age. You may quote that passage from the New Testament which says: " If thy right hand offend thee, cut it off and cast it from thee"; but you must remember, that it was only to be done when the whole body was in danger of being cast into hell fire. In our profession I am satisfied, that anesthetics for tooth extraction have proved more of a curse than a blessing. It is like the wholesale use of morphine to escape pain. I remember about eighteen years ago I broke down under great excitement and excessive labor during the war. Came down with sciatic pains and was advised by a good physician and a good friend, to use morphine as a relief. As I began I found a nervousness got hold of me such as I had never experienced before, and was told to double the dose and I would be all right. When I did this I found the cry for more sounding in my ears like so many famishing men; and having read De Quincy's confessions of an opium eater many years before, I abandoned my pills and my office for nearly three months, threw away my instruments, went into the fields and lived nearer to nature and my health was restored. The law of gravitation is always at work among professional men to drag them down; but you will remember that there is a principle in the little acorn that pushes itself high in the air against all this, and it becomes a mighty oak, and this corresponds to the moral force in man. Health is the *rule;* and disease is the exception, though we see it on every hand. If it was not for the bother and encroachments on our time, it would be better that we have no fee for tooth extraction, lest we be led into temptation by the love, or the need of the almighty half-dollar. It would be as consistant for the physician to throw away his medicine and practice surgery alone; in order that he might skilfully amputate the limbs rather than to cure them: as to extract the teeth in a majority of cases that present themselves to the ordinary practitioner in dentistry, and this is the foundation of reform in our calling.

Systemic treatment and local, for constitutional difficulties, is to be the future of our profession. The people must be well informed on this subject: and all shams will pass away as light illumines the mind. Beware how you receive truth; is a maxim that belongs to the past. Ignorance is the pestilence that has followed the race in every age of the world. The epoch of Fetishes is fast passing away. If the Bibles and liturgies and homilies are consecrated to truth the world has nothing to fear. Shams will disappear, mysticism will go into the dark where it belongs: and guilt and hypocrisy will be so transparent that we shall be forced to abandon it in every profession, as we are when we deal with nature. What good farmer would dream of acting the hypocrite with a hill of corn or potatoes? or in harvesting his grain or of his treatment to his animals in the stables? as men often do to their neighbors, or their wives and their children. Let it be once thoroughly understood, that there is no escaping punishment; and we shall learn to be sincere and just and honest to the world. This nature that surrounds us on all sides and of which we make the human part, is continually knocking at the heart and intellect of man from the moment he draws his first breath of life until its close. And we treat her admonitions as we do peddlers who come to our doors to vend their wares. We bid her begone; her errand she is hardly allowed to state, however necessary, urgent or profitable. Thought is only a manifestation of nature in her thousand ways of communication.

It is flitting as the sunlight amid the clouds in the thunder storms, and it can never become valuable until it takes form and assumes a stated fixedness in the mind, and then it becomes an active factor in man's daily life. When this condition of things exists, we cannot help talking if we would; and to "do good and to communicate" will be a part of our very being. Enthusiasm in goodness is harmless, and there is little danger of a superabundance in the world; it is far better than stagnation and death. Enthusiasm is indispensable to help undo the errors, as well as to plant the truth; the weeds must be destroyed as well as the plants cultivated, and the weed of the most luxurious growth of to-day in our profession is tooth extraction.

In the December number of the Cosmos is an article on "The present Status of Dentistry in the Country," by a namesake of mine who resides in California, in which the gentleman goes on to state that the average duration of gold fillings (and he believes gold to be the best,) is only about five years, now, if this is true, our profession is a failure. Since I began this paper, I have had calls from four different persons, *married ladies*, whose teeth I have had charge of for nearly twenty years; neither of them has lost a tooth in all these years, though some of them have had to be refilled, and a few of them more than once. I have also refilled two teeth that were filled only four years ago, but these two were the only ones out of thirteen cavities filled at that time, and one of the two had a fracture in the enamel that weakened it, and all the others were as bright and clear as when I left them and no discoloration whatever, though one was a large contour filling.

All these persons have families, and the last mentioned has a child nearly two years old and has been under treatment from her physician a good share of the time since it was born. I think the average of good gold fillings is more then ten years, and if that is true, dentistry is the cheapest commodity in the market for what it costs in money. I have noticed that the most difficult operations have always lasted the longest with myself, which is good evidence that those that have failed were imperfectly done, which I believe to be the almost invariable rule. I have observed also that more failures are found at the cervical wall and under the free margin of the gums; and that a large portion of the failures in this direction are caused by excessive gold, that dams up the mucus and saliva and causes a lodgment of food that hastens decay. We find also that teeth filled with gold do not show signs of failure in every portion of the cavity at the same time, while in amalgam fillings, the discoloration is nearly universal and extends around the borders of the enamel in all portions nearly equal. I have not found any incompatibility between gold and enamel or tooth-bone as is suggested by some writers; but the incompatibility is between the hand, the heart and the instrument of the man who performs the operation. The man is out of place as well as the filling. The incompatibility lays between the man and his profession.

Almost all our literature for the past few years, while it has tended greatly to elevate our profession as to immediate and remote causes of the decay and loss of the teeth, has somewhat neglected the subject of tooth extraction; and I have dwelt thus long upon this subject, because it lays at the foundation of all our difficulties as a profession. It is the worm that is gnawing at the root, that will crumble it to atoms and admit within our borders all the destroyers of our peace. Heretofore, the best methods of extracting the teeth have commanded the attention of the profession; but the work of to-day is to learn the best way how not to extract teeth at all. This is the Battle of the Wilderness. We may take Vicksburg and Shilo and Donaldson, but without this Battle of the Wilderness we shall never have peace. When this is done, we shall hear no more about cheap dentistry or dear dentistry, but our profession will stand upon its merits like the other professions. We shall be no longer pullers down, but *doctors indeed.* Turn the war-fare in this direction; talk of it in the school-houses or in any other place if you will; I am aware that I am treading on consecrated ground when I declare it is in the fullest harmony with a profession to talk whatever truth it has to the world, providing we do not exaggerate and boast of being able to perform more than we can do.

To educate the masses is part of the individual, as well as the collective duty; and who shall say that any truth shall not be proclaimed upon the house top, or in the street, or through any medium within our reach. In an age like our own nothing is consecrated that will not bear the severest criticism. The consecration of Emperors, Kings, Bishops, Cardinals and Priests is fast passing away, all the sciences are freely talked, all political and religious convictions are sounded from school-houses, as well as colleges. What we need is to enlighten the people in the laws of Physiology and Hygiene, and I can see no more impropriety in talking of it in any proper place, than in talking about it in the office. In these days of railroads every thing is done in this way. And where the State makes it the duty to educate its children, there can be no good reason why the masses should not be enlightened by any means within our power. If it is a new departure, no man has a right to stand by in churlish indifference to the progress of his age. There is nothing necessary to become a good dentist, but *adaptation* and *consecration.* From the very moment the mother hushes up the cry of the new born infant, up to the life of the statesman, the hero or conqueror; and so down through all animal and vegetable life, and then back again to the creator of the universe, there is nothing but *adaptation* and *consecration.*

I cannot forbear saying at this stage of the discussion of the subject, that all persons who are parents and have the means within their reach and who do not see to it that the teeth of their children are preserved if possible, are *criminals.*

They may excuse it by saying they have no faith in it; but that only makes it so much the worse; for the means of information are within the reach of every one, and we have no right to decide upon any subject until we have investigated by every means that can be obtained. The children are not only injured who are the innocent victims of their existence, but the parents are injured. Everything grows from within and everything grows by *reaction.* It is the same with all animal life from the first pulsation of the heart in human life down to the smallest flower by the road side; and every act of selfishness weakens the pulsation of our existence. We only touch nature at the circumference; as we touch all blossoms and plants, and we may grind to powder every particle of matter in the world and we only reach the circumference of each separate thing. If it were different, we should be infinite. So as the trees and plants and blossoms decay, they begin at the circumference and life diminishes toward the center by an invariable law; and this is the foreshadowing of immortality; "for as the dust returns to the earth as it was; the spirit returns to the God who gave it."

It does not require genius as much as constancy to be an acceptable dentist. There is nothing in fact to genius but the power of constant effort and consecration. And I am here to confess that for the first ten or twenty years of practice (although I shall never be accused of lack of enthusiasm), I did not arrive at the true conception of the possibilities of saving the teeth that I feel to-day. I think it was Rubens, the painter, who said he had just begun to improve when he had reached his ninety-third year. The crowning objection I shall urge against extracting teeth is, that it destroys the unity of the whole universe of which man is the microcosm.

But I think I hear the objector saying: "You are mistaken, everything is dual;" "we have the poles as well as the equator." But the poles are only the imaginary points; the mistakes and errors, upon which the whole universe of truth and love is rolling, that will finally crush out all the false that obstruct our progress to a higher and more perfect civilization. The great benefit to be derived from the new departure is, that it will stimulate the dentist with the hope and possibility of saving every tooth. Artificial crowns are also a step in the right direction, made of gold which is always the best, but which can easily be adjusted with amalgam. Inserting artificial teeth over the roots of teeth that are sound, is another advance, especially in partial lower sets: I know of many that have been worn fifteen or twenty years without inconvenience, that have been far more useful than where the roots have been extracted. I remarked near the commencement of this paper that Thought was a manifestation of nature: let us see what nature gives us to prove this assertion. America has rightfully been called the New World, and the American man a New Man, made up of all nationalities, born into freedom; he is as free as the soil on which he is born. He is touched by nature on every side and in every step in his life and it becomes in some way manifest to his senses.

He cannot go beyond nature with his thought. When he has observed all that can be seen with the telescope and the microscope it is space and is infinite. Nature is a sphere, the sun a perfect sphere, the earth an imperfect sphere and everything upon the earth tending in that direction. Man as a whole, or in his several parts: the head, the eyes, the nose, the mouth, the heart, the liver, the lungs, the intestines and every particle of which they are composed, the blood corpuscles when filled with oxygen, those little errand boys of nature that carry the fuel to warm and keep alive the whole body are all spherical; also the lines of commerce upon which they do their errands. All insect life the pebbles, the dust, all liquid bodies, all crystallization as we find them now or when resolved into their original state. All egg life, all embryo life, the whole tree with its spreading branches, all the leaves, the buds, all the flowers, all the odor of the flowers is distributed in the spherical form. The vegetables, the fruits, the protoplasm and all cell life, it is all the same from the atom to the sun.

The logic of all this is, that if thought is a manifestation of nature, thought is spherical. If nature is a sphere, thought must be spherical if it is a manifestation of nature If our hypothesis is not right, let us be as honest as the scientists are, and remove the hypothesis. We all confess that the sphere is infinite, while we are finite. If this will not bear the test of scrutiny, let us use it as an illustration in climbing. The sphere being infinite and we finite, we only see: or it manifests itself to us only in that portion that is within the vision of our senses. All barbarous nations live near the base; with savage beasts and venomous reptiles to correspond. A little higher up are the semi-barbarous, like the North American Indian, with the buffalo, the bear, the fox and the wolf, to correspond to their civilization. A little higher still is our own civilization, with the horse, the cow, the dog and all the domestic animals that correspond to our own civilization, from the most refined to the most doubtful.

With all the paintings and music and flowers and dress and emotions that are only these thoughts in action; that produce all our philanthropics and institutions of learning with our bibles and creeds as perfect as nature has so far communicated to us: and we are not yet up to the meridian line. A little higher up are the philosophers, who have higher manifestations than mere creeds; and higher still the scientists, who overlook the whole because they have higher manifestations and can see more, and are only climbing to arrive at the highest truth in nature and so up to the infinite, if possible with our finite capacities. If this is true, it will account for all our bickerings and disputes; and if we accept this, we can pity and apologize and forgive all the short comings in this generation and only beg of them to come up higher till we all see alike, and dwell in fullest harmony and peace.

Did you ever think friends that it may be possible that the animals in corresponding to the civilization of man feed and sustain their lives on the barbarisms and passions that are within us: and that they exist by an inexorable law: they are part of the ultimates of creation: and that they cease to exist by that same inexorable law, as we become pure: and that *that* is the reason persons who are pure instinctively shrink from the presence of the vile? That fact once established solves the problem of delirium tremens. When we are debauched enough, the snakes and devils come back and take up their abode with us and have a state of fixedness in our very being. That our depraved natures grow more depraved by living on impure thoughts and that is the reason the law is made to suppress all vile literature. If we have come up through all the evolutions that many suppose from the lowest animal life to our present state perhaps it is true that there are the remains of that low state in us: and as we retrograde, we fall back into the lower orders of creation in our hearts: and by continuing in sin revive the passions and deadly elements that belong to a lower order of animal life.

In the different stages of manifestations in our mistakes from self derived intelligence or imperfect manifestations, our very freedom leads to excesses and license.

Our great civil war showed the necessity in this spherical order in the government of our country. The blood and treasure it cost showed we had lost that roundness and perfection that belongs to all good government. And the ax of civilization had to be laid to the roots of secession and error, to lop off the rough corners, so that our government would roll through the years without clogs or hinderances.

Why is it I ask, that medical men and the dentists to a great extent are charged with materialism, without it is by the inductive reasoning insisted upon in the school studies. The nearer we live to true nature and the higher her manifestation to us, the more devout and really religious we ought to become. There are many thoughtful persons who sincerely believe we have reached the highest point in our civilization and are now beginning to decay. These men point to the civilization of Greece and Rome as an illustration and can see a perfect parallel between them and ourselves when they began to go down: and charge their fall to their infidelity. But when I see some young ladies in the dental class learning our profession with their quick insight or inspiration that always reaches things by deductive reasoning and feel that universal suffrage is soon to be extended to every person without regard to sex, and then remember that not more than one fourth of the citizens of ancient Rome were allowed to vote, and Buckle tells us that in the four hundred years between Homer and Plato, while the Greeks made great improvements in arts and in practical life; and that through all these years the women actually lost ground, that the civilization was but half a civilization: that the women were *things* and mere *toys*, while to-day they are helpers and soon to be equals in every department of life; I am satisfied that woman made the equal with man, we cannot fail without the *divine government* fails, which cannot take place. Why, my friends, you might as well try to revolve a sphere on a single segment, *which is no sphere at all*: and have a nation exist, as to think it can last without the equality of the sexes; and it is because we have lived so near the base of the sphere and have got such low manifestations of nature, that the masses have failed to see the highest truths; and done injustice to woman so long.

Buckle tells us of experiments in the mineral kingdom in crystallization, that excited the attention of Philosophers, who tried to discover their secrets by *induction*. Men, who broke up the crystals; measured and weighed them, and subjected them to all manner of experiments for more than twenty years, and were baffled in every way until they were worn out. When a Frenchmen, a poet, by the name of Hauy, saw by his poetic vision that these crystals were perfectly regular in form; and their secondary forms deviated from their primary forms by a regular process of diminution; by what he called the law of decrement; the principles of decrease being as unerring as those of increase. The poor laborers of the past year saw in the same way, when they advocated the paper money system; they saw that there was no real value to a dollar only in the relation it bears to labor.

Wendel Phillips tells us of the lost arts: but the greatest art was lost to aristocrates when the deductive reasoning began and the scholastic reasoning was crushed out, by which they made slaves of men and women. Swedenborg proclaimed deductive reasoning when hesaid all force is spiritual force: and that our natural bodies are the ultimate forms of life, and that the true order of reasoning was from the center to the circumference. When we are elevated enough to receive the highest manifestations of nature into true manhood, we must go forward we cannot go backward. Manifestations of nature are nothing until we incarnate them in our lives. Man is the representative of the understanding principle says Swedenborg, and woman is the representative of the *love* principle and the two must be blended together into one, to make perfect our life, our institutions and our country.

Moses had a glimmer of the deductive method, when he said the Lord showed mercy unto thousands who loved him and kept his commandments. Some of the Prophets had it, when they predicted the sure consequences of sin and the certainty of punishment for a violated law. Every person has this deductive method who adjusts the coarser principles of barbarism to a higher civilization or a more beautiful life. Newton reasoned in the same way, when he saw the apple fall from the tree, and sat still in his chair till he saw the law of gravitation. Goethe saw it when he saw that pistles, corollas and petals of plants were only modified leaves. Edmund Spencer saw the same thing when he wrote:

> " So every spirit as it is more pure, and hath in it more
> of the heavenly light;
> So it the fairer body doth procure to habit in.
> For of the soul the body form doth take; for soul is
> form and doth the body make."

What has made Mr. Emerson, what he is, but these same manifestations of Nature, when he strolls into deep ravines in the early morning on foot and alone; with his thought-book in his hand or pocket to note the budding of some wild flowers that are late in opening their eyes in the shady nooks of the woods, or going abroad in the storms; so these manifestations of Nature shall have no obstructions made by the hand of man. In his long life of devotion to Nature the very blood in his veins has become finer, his flesh is so pure that he is transfigured every morning with the rising of the sun. He has lived to see his thoughts incarnated into all peoples, kindreds and tongues, his face is radiant with beauty, and his smile is the most glorified smile I ever beheld, and he is nearly eighty years old. If you would reach this high seat, young friends, go on climbing in your profession, in work and in worship, go climbing on your knees if it is necessary, and remain there until the world bids you take a high seat in her Temple.

