# Correction

**Published:** 1880-06

**Authors:** 


### CORRECTION.

In the May number of the *Register*, by some oversight of the printers, two pages—one leaf—was omitted. It occurs in Dr. Robinson's address, between pages 212 and 213, two pages belong between these. The leaf will be sent out in the present—the June—No., marked page 212 a and 212 b, thus it can easily be placed where it belongs by all who have both numbers, and by the binder. "Mistakes will occur, &c.," says the printer.

